# HEMA-free versus HEMA-containing adhesive systems: a systematic review

**DOI:** 10.1186/s13643-025-02763-w

**Published:** 2025-01-21

**Authors:** Esraa Abdelkhalek, Hamdi H. Hamama, Salah H. Mahmoud

**Affiliations:** Conservative Dentistry Department, Faculty of Dentistry, Mansoura University, Mansoura, Postal Code 35516 Egypt

**Keywords:** HEMA, Dental bonding, Bonding durability, HEMA-free, Systematic review

## Abstract

**Background:**

Hydrophilic monomer 2-hydroxyethyl methacrylate (HEMA)-free adhesive systems are gaining increasing popularity nowadays. Although the addition of HEMA to dental adhesives improves dentin wettability and resin diffusion into demineralized collagen fibrils, HEMA’s high hydrophilicity can lead to hydrolytic degradation of the adhesive interface. Thus, HEMA-free adhesive systems have been developed. Unfortunately, the lack of HEMA in the adhesive composition may lead to a separation phase between hydrophobic and hydrophilic components. The aim of this systematic review was to evaluate the clinical performance of HEMA-free adhesive systems and compare them with HEMA-containing ones.

**Methods:**

An electronic search of The National Library of Medicine (MEDLINE/PubMed) was conducted. Eligibility criteria were reporting empirical data from clinical studies published between 2013 and 2023 about the clinical performance of HEMA-free adhesive systems for direct resin composite restorations. Studies with at least 2-year clinical follow-up done in permanent dentition in any form of cavities were selected. The included studies were assessed for risk of bias using the modified Cochrane Collaboration tool criteria.

**Results:**

The database search returned 147 studies; a total of 7 studies were included in this review; the majority of studies reported no significant difference between the two types of adhesives for the parameter of retention.

**Conclusions:**

HEMA-free adhesive systems exhibited good clinical performance with regard to retention. There was some concern about their influence on marginal adaptation and marginal discoloration due to the conflicted results reported by the included trials. Thus, the results need to be confirmed with long-term evaluations.

**Systematic review registration:**

PROSPERO CRD42023448952.

## Introduction

Obtaining strong adhesion to the moist dentin remains clinically challenging due to substrate heterogeneity and hydrophilicity [1]. Therefore, in self-etch adhesives, dentin priming is often promoted by hydrophilic monomers such as 2-hydroxyethyl methacrylate (HEMA) to improve dentin wettability and resin diffusion into demineralized collagen fibrils. Moreover, it acts as a diffusion promoter for other monomers to form the hybrid layer [2]. Unfortunately, HEMA’s high hydrophilicity can lead to some long-term disadvantages, such as increased water uptake and hydrolytic degradation of the adhesive interface [3, 4]. This can lead to some clinical problems such as dentin sensitivity, marginal discoloration, and possible caries recurrence. These problems compromise the longevity and performance of the restorations [5].

In order to avoid the negative effects of HEMA monomer, adhesive systems without it have been developed, called HEMA-free adhesives. However, removing HEMA from the adhesives may increase the risk of a phase separation reaction between hydrophobic and hydrophilic components [6, 7]. The phase separation of HEMA-free adhesive would produce water droplets within the polymerized adhesive layer, frequently expressed by water-tree nanoleakage. The water droplets appear in the resin/dentin interface due to osmotic infiltration of remaining water on dentin subsurface or being transported from dentinal tubules [1, 6].

Considerable debate exists on the clinical performance of HEMA-free adhesives. Some studies [8, 9] reported different clinical performance between HEMA-free and HEMA-containing adhesive systems. Conversely, other studies [10, 11] showed no significant difference between the clinical performance of the two adhesive systems. The systematic review by Silva et al. [4] reported that HEMA-free adhesive systems did not have better clinical performance than HEMA-containing adhesive systems in NCCL restorations. This review by Silva et al. [4] had some limitations. First, it included old studies from the 1990s before the introduction of universal adhesive systems, because modern universal adhesives were introduced during the 2010s [12]. Although Kuraray released Clearfil SE Bond (Kuraray Noritake Dental, Tokyo, Japan) in 1991 which is considered the gold standard in this category, it did not reveal about the adhesive’s component or the discovery of 10-MDP. After Kuraray’s patent on 10-MDP expired, 3M Oral Care launched Scotchbond Universal Adhesive in 2013 and since then many manufacturers have followed and developed universal adhesive systems [13]. Second, the systematic review by Silva et al. [4] included studies with only one type of cavity design. Unfortunately, adhesion to dentin affected by non-carious cervical lesions (NCCLs) has a high percent of adhesive failure, which may compromise the longevity of restorations. This is due to molecular and chemical structural changes at the interface leading to less favorable adhesion [14].

Therefore, this current systematic review aims to evaluate the clinical performance of HEMA-free adhesive restorations in the previous clinical trials, and summarize the findings from these trials about the durability and longevity of HEMA-free adhesives.

## Methods

The protocol of this systematic review was designed following the Preferred Reporting Items Systematic Review and Meta-Analysis (PRISMA) guidelines [15, 16]. The protocol was registered in an international database of prospectively registered systematic health and social care reviews (PROSPERO) systematic review number: PROSPERO CRD42023448952. The research question based on the PICOS [17] strategy was as follows: Do HEMA-free adhesive systems have better durability than HEMA-containing adhesive systems clinically? Defined as.

- P (population)

The review will include patients with permanent dentition in need of restorations.

- I (intervention)

The review will include composite restorations placed with HEMA-free adhesive systems.

- C (comparison)

The review will include composite restorations placed with HEMA-containing adhesive systems. O (outcome).

The outcome of this review will be the clinical performance and the durability of restorations from functional, esthetical, and biological aspects.

From functional aspect, the retention rate will be assessed as the proportions of the restorations retained in the lesions. Marginal adaptation will be measured as the defect of the margin that can be felt when moving a sharp probe over the restoration margins.

From esthetical aspect, marginal discoloration will be measured as the discoloration along the restoration margins that can be detected visually after air-drying the tooth and after removing plaque. Colour match will be assessed as the mismatch in colour, shade, and/or translucency between the restoration and the adjacent tooth structure.

From biological point of view, post-operative sensitivity will be assessed as the combination of thermal or tactile sensitivity after air blowing and moving a probe over the lesion/restoration. Caries occurrence along the restoration margins or underneath the restoration can be detected visually and tactilely using a probe after air-drying the tooth. Finally, tooth vitality can be tested using thermal sensitivity tests.

- S (study type)

Randomized clinical trials and clinical follow-up studies will be the types of studies included in this review.

### Eligibility criteria

For eligibility criteria, the included studies were assessed for the following inclusion criteria: randomized clinical trials (RCTs) comparing the clinical effectiveness of HEMA-free and HEMA-containing adhesive systems for direct resin composite restorations in permanent dentition in any forms of cavities (Black’s class I, II, V) and non-carious cervical lesions (NCCLs). Moreover, studies with adult patients (male and female) of any age group, studies with at least 2-year clinical follow-up, parallel or split-mouth studies, and studies done between 2013 and Dec 2023 were included. There were some exclusion criteria: studies published before 2013, non-English language manuscripts, restorations were done on primary or endodontically treated teeth, and clinical studies without follow-up. Furthermore, editorial letters, pilot studies, historical reviews, literature reviews, systematic reviews, in vitro studies, cohort, and observational and descriptive studies, such as case reports and case series, were also excluded.

### Data sources

The following database was searched for studies published between 2013 and 2023 that reported on the durability of HEMA-free adhesive systems: The National Library of Medicine (MEDLINE/PubMed). The keywords used were as follows: (“bonding durability” OR “dental bonding agent” OR “clinical performance” OR “composite restorations” AND “HEMA” OR “HEMA-free” OR “HEMA-containing”). Articles that met the inclusion criteria were imported into EndNote X7 software. The studies were further checked manually to find additional studies that were not identified through the search of the electronic databases.

### Study selection

The selection of studies went through three stages: selection according to the title, the abstract, and analysis of the full text. All retrieved studies from the searches were reviewed by each review author against the review’s eligibility criteria, and consensus was achieved on their inclusion. Each eligible study received an ID combining the first author’s name and year of publication.

### Data extraction and synthesis

One reviewer independently extracted the data from the included studies and assessed risk of bias. Data that was extracted was tabulated and listed, and the extracted data and risk of bias assessments were corroborated by a second reviewer. When disagreements arose, these were resolved by consulting a third reviewer. If any relevant data was missing from a paper, the corresponding author of this paper was contacted by e-mail.

### Risk of bias assessment

The Cochrane Collaboration’s tool [18] for assessing risk of bias in RCTs was used to perform the quality assessment of the trials, which included the following domains: selection bias (randomization, allocation concealment, unit of randomization issues), performance bias (blinding of participants, operators, examiners), detection bias (blinding of outcome assessment), attrition bias (loss to follow-up and missing values or participants), and reporting bias (unclear withdrawals or reported outcomes). We did not have access to study protocols, so selective outcome reporting was not assessed in this study. Bias was assessed as a high, low, or unclear judgment. RevMan 5.4 (ROB1 tool) (RevMan 5.4, The Nordic Cochrane Centre, The Cochrane Collaboration, Copenhagen, Denmark) was used to obtain a risk of bias summary and graph for the selected studies [18].

At the study level, sequence generation, allocation concealment, and blinding of the outcome assessment were considered the key domains for the assessment of the risk of bias. To be considered at “low” risk of bias, studies should present low risk of bias in all key domains. If one or more domains are judged to have unclear risk, the study was judged to have unclear risk. If at least one study is considered at high risk of bias, the study was considered to have a high risk of bias.

## Results

### Search details

**Fig. 1:**
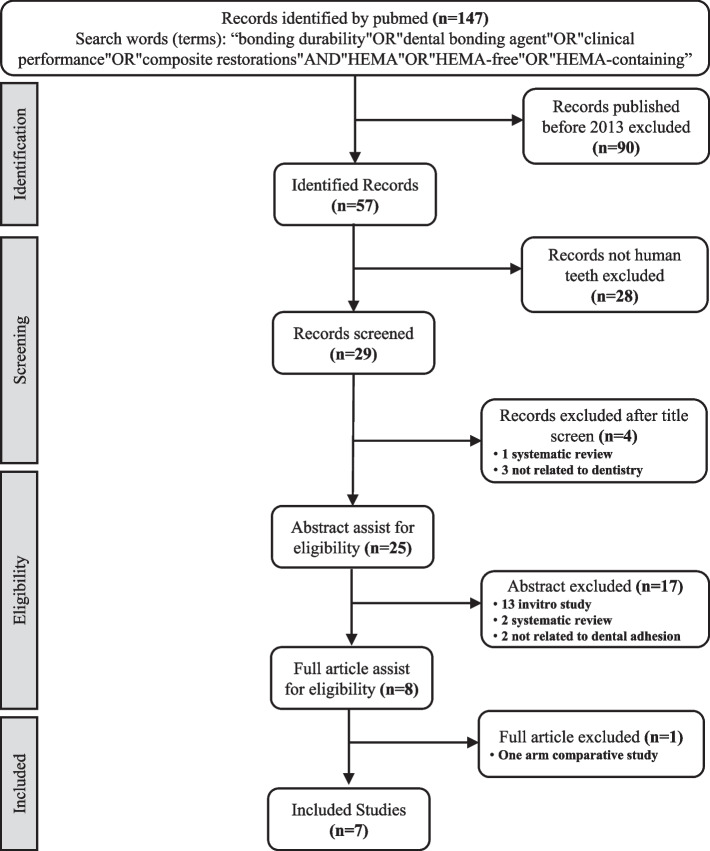
Flowchart showing the study selection process based on PRISMA statement

The search identified 147 studies; after eliminating the duplicates and studies published before 2013, 57 studies were finally identified. Twenty-eight studies were excluded as they were not done on human teeth. Therefore, 29 records remained. A total of 6 of these studies were excluded after title evaluation. After evaluating 23 remaining studies, seven of the studies were included in this systematic review. The details of the selection process are illustrated in the flow chart shown in Fig. 1.

### Study characteristics

The selected studies were published between 2013 and 2023. The material, objectives, and conclusions of each study are summarized in Table 1. The methodological assessment of the included studies included evaluation of the trial design, evaluation criteria, sample size, material selection, restoration type, isolation, follow-up, and recall rate which are listed in Table 2.

**Table 1:** Summary of studies included in systemic review

Study\ country	Year	Materials	Objective	Conclusion
(1) Morett et al. [19] Brazil	2013	-HEMA-free adhesive G-Bond (GC, Tokyo, Japan) -HEMA-rich Clearfil 3S Bond (Kuraray, Tokyo, Japan) -composite Gradia Direct Anterior (GC, Japan)	To evaluate the 3-year clinical performance of two one-step self-etch adhesives, namely one HEMA-free and one HEMA-rich system, in non-carious cervical lesions (NCCL)	Both one-step self-etch adhesives presented an equally favorable clinical effectiveness at 3 years follow-up
(2) van Dijken [23] Sweden	2013	-HEMA-free 1-step SEA adhesive G-Bond -hybrid resin composite Gradia Direct posterior (GC, Tokyo, Japan) -HEMA-containing 2-step SEA adhesive FL-Bond adhesive -giomer resin composite Beautifil (Shofu, Kyoto, Japan)	To investigate the clinical longevity of class II restorations performed with a HEMA-free1-step SEA adhesive in combination with a micro-fine hybrid resin composite. The restorations were compared intraindividually with Class II HEMA-containing 2-step SEA – giomer restorations	According to the durability in Class II cavities, the HEMA-free adhesive was successful after 6 years. The HEMA-containing SEA-giomer restorations showed rather high failure frequency
(3) van Dijken [23] Sweden	2013	A single-step, self-etching HEMA-free primer (G-Bond, GC Corp, Tokyo, Japan), a 3-step HEMA/TEGDMA free etch-and-rinse (cfm, Saremco AG, Rebstein, Switzerland) and a 2-step HEMA-containing etch-and-rinse adhesive (XP Bond, Dentsply/DeTrey, Konstanz, Germany) were evaluated in combination with two restorative resinous materials, composite Gradia Direct (GC Corp) was used in combination with G-Bond, and els (extra low shrinkage; Saremco)in combination with the two other adhesives	To determine the long-term clinical bonding durability of a 1-step HEMA-free SEA, a 3-step HEMA/TEGDMA-free etch-and-rinse, and a 3-step HEMA-containing etch-and-rinse adhesive in Class V non-carious cervical lesions without using retention of external lesion surface area	It can be concluded that the durability of non-carious cervical lesions of the HEMA-free adhesives was successful after 5 years. Despite concerns which have been raised, the 1-stepSEA showed one of the best clinical dentin bonding effectiveness
(4)Van Landuyt et al. [9]	2014	1-step SEA G-Bond (GC, Tokyo, Japan). 3-step E&R Optibond FL (Kerr, Orange, CA, USA) Micro-hybrid composite Gradia Direct (GC, Tokyo, Japan)	To determine the long-term clinical performance of a HEMA-free 1-step SEA. This randomized controlled trial was set up to compare the clinical effectiveness of a HEMA-free 1-step SEA with a three-step etchant-rinse adhesive	Restorations bonded with G-Bond did not need clinical intervention more often than those placed with the 3-step E&R Optibond FL and had a high retention rate (> 90%) after 5 years of clinical service. There were, however, differences between both tested adhesives, not decisive for the clinical success rate. The HEMA-free one-step self-etch adhesive exhibited significantly more enamel defects and stains. In the majority of cases, these defects were insignificant, but after 5 years, several restorations also failed because of deep micro leakage
(5) Peumas et al. [20] Belgium	2018	-HEMA-free 1SEa G-Bond (GB; GC, Tokyo, Japan) −3E&R Optibond FL (OFL; Kerr, Orange, CA, USA) -Microhybrid composite (Gradia Direct GC)	To evaluate the 9-year clinical efficacy of the HEMA-free 1SEa G-Bond compared to that of 3E&Ra Optibond FL	Class-V restorations bonded with the HEMA-free 1SEa performed clinically equally successful as those bonded with the 3E&Ra. However, more marginal deterioration, though still clinically acceptable, was recorded at the incisal enamel side for the 1SEa
(6) Tekce et al. [21] Turkey	2018	G-aenial Bond (GC Corp, Tokyo, Japan) -Clearfil S3 Bond (Kuraray.Medical Inc, Tokyo, Japan) -Fortify Plus Filled Surface Sealant (Bisco, Schaumburg, IL, USA) -Clearfil Majesty Posterior Kuraray Medical Inc, Tokyo, Japan)	To evaluate the clinical performance of HEMA-containing and HEMA-free all-in-one self-etch adhesives with and without a surface sealing process in Class I cavities	None of the restorations failed after 2 years, and there were no significant differences between the clinical performance of HEMA-containing and HEMA-free all-in-one self-etch adhesives with and without surface sealing in Class I restorations over the same time period
(7) Oliveira et al. [22] Brazil	2023	*-*Prime & Bond U *(*Dentsply Sirona, Konstanz, Germany) *-*Optibond All-in-One* (*Kerr, New South Wales, Australia) *-*Clearfil SE* (*Kuraray Medical Inc., Tokyo, Japan) -Filtek Z350 XT Color A3 composite resin (3 M ESPE, Sumar´e, SP, Brazil)	To evaluate the clinical performance of a self-etching adhesive system associating the functional monomers HEMA and 10-MDP in the restoration of NCCLs over 2 years	The association of HEMA and 10-MDP monomers in the self-etching adhesives did not influence the Clinical performance of the NCCL restorations with respect to retention, post-operative sensitivity, and incidence of secondary caries. However, positively influenced the marginal adaptation and marginal staining at the 2-year follow-up

**Table 2:** Methodological assessment of included studies

Study ID	Study design	Population			Intervention group		Control group		Outcome		Recall period	Recall rate
		**No. of** **patients and gender**	**No. of restorations and type of cavity**	**Mean range of** **patients**	**HEMA-free**	**Success rate** **or AFR**	**HEMA-containing**	**Success rate** **or AFR**	**Evaluation criteria**	**Evaluation parameters**		
(1) Moretto et al. [19] 2013	RCT Parallel	Total = 30 Male = 12 Female = 18	175 NCCLs	20–69 years	G-Bond (GC, Tokyo, Japan)	97.6%	Clearfil 3S Bond (Kuraray, Tokyo, Japan)	92.6%	Vanherle criteria [24]	Retention, marginal adaptation Marginal discoloration, secondary caries, post- operative sensitivity tooth vitality	3 years	96.7%
(2) Van Dijken [23] 2013	RCT Split mouth	Total = 54 Male = 30 Female = 24	115 Class II	24–77 years Mean = 57.1	G-Bond (GC Corp, Tokyo, Japan)	91.5%	FL Bond 2-step SEA (Shofu, Kyoto, Japan)	82.3%	Modified USPHS	Anatomical form, marginal adaptation, marginal discoloration, color match surface roughness, caries, post- operative sensitivity	6 years	96.5%
(3) Van Dijken [8] 2013	RCT Split mouth	Total = 67 Male = 34 Female = 33	169 NCCLs	39–84) years Mean = 64.7	A single-step HEMA-free G-Bond (GC Corp, Tokyo, Japan)	nr AFR = 1.6%	(XP Bond, Dentsply/ DeTrey, Konstanz, Germany	nr AFR = 5.4%	Modified USPHS	Retention, marginal adaptation, marginal discoloration, secondary caries, post- operative sensitivity , color change, surface	5 years	94%
					−3-step HEMA/TEGDMA free etch-and-rinse (cfm, Saremco AG, Rebstein, Switzerland)	nr AFR = 1.7%						
(4) Van Landuyt et al. [9] 2014	RCT Parallel	Total = 52 Male = nr Female = nr	267 NCCLs	nr	G-Bond (GC, Tokyo, Japan)	87.4%	Optibond FL (Kerr, Orange, CA, USA)	90.9%	Vanherle criteria	retention, marginal integrity, marginal discoloration, caries occurrence, sensitivity	5 years	86.5%
(5) Peumans et al. [20] 2018	RCT Parallel	Total = 52 Male = nr Female = nr	267 NCCLs	nr	1SEa G- Bond (GB; GC, Tokyo, Japan)	80.3%	3E&Ra Optibond FL (OFL; Kerr, Orange, CA, USA)	79.5%	Vanherle criteria	Retention Marginal integrity Marginal discoloration Caries post-operative sensitivity	9 years	82.6%
(6) Tekce et al. [21] _2018_	RCT Split mouth	Total = 40 Male = 15 Female = 25	160 Class I	18–55 years Mean = 23.3	G-aenial Bond (GC Corp, Tokyo, Japan)	100%	Clearfil S3 Bond (Kuraray Medical Inc, Tokyo, Japan)	100%	Modified USPHS	Retention, marginal adaptation, marginal discoloration, secondary caries, color match, loss of anatomic form, surface	2 years	87.5%
(7) Oliveira et al. [22] 2023	RCT parallel	Total = 17 Male = 8 Female = 9	60 NCCLs	23–39 years Mean = 30	Prime & Bond U (Dentsply Sirona, Konstanz,Germany)	nr	-Optibond All-in-One( Kerr, New South Wales, Australia)	nr	Modified USPHS	Retention, marginal adaptation Marginal staining, post- operative sensitivity Secondary caries	2 years	96.6%
							*-*Clearfil SE* (*Kuraray Medical Inc., Tokyo, Japan)					

*RCT* Randomized clinical trial, *nr* not reported, *NCCLs* Non carious cervical lesions, *AFR* Annual failure rate

### Risk of bias assessment

The assessment of the “risk of bias” in the included studies was performed and is presented in Table 3. The risk of bias was low in the majority of domains; random sequence generation and allocation concealment were reported in all seven studies. There were several methods of randomization used in the included studies, such as randomization tables [19, 20] and flipping a coin [21]. Oliveira et al. [22] used the Random Allocation 2.0 software. The remaining studies did not mention the method of randomization [8, 9, 23].

**Table 3:** Risk of bias assessment

Study ID	Random sequence generation(selection bias)	Allocation concealment (selection bias)	Blinding of participants and personnel (performance bias)	Blinding of outcome assessment (detection bias)	In complete outcome data (attrition bias)
Moretto et al. [19] 2013	Low	Low	Unclear	Low	Low
Van Dijken [23] 2013	Low	Low	Unclear	Low	Low
Van Dijken [8] 2013	Low	Low	Unclear	Low	Low
Van Landuyt et al. [9] 2014	Low	Low	Unclear	Low	Low
Peumans et al. [20] 2018	Low	Low	Unclear	Low	Low
Tekce et al. [21] 2018	Low	Low	Unclear	Low	Low
Oliveira et al. [22] 2023	Low	Low	High	Low	Low

Blinding of participants and personnel was unclear in six studies [8, 9, 19–21, 23] and high risk in one study [22]. It was obvious in the included studies that blinding of dental operators was not possible as they need to know the application of the adhesive system used. Moreover, it was not possible to blind the patients in this study [22] because one of the adhesive systems followed a two-step application process and the others a single-step application process. The clinical evaluators in all studies were blinded so detection bias was low. The attrition bias was low because all studies adequately reported the number of dropout. Therefore, these studies [8, 9, 19–21, 23] considered unclear risk of bias, but Oliveira et al. [22] considered high risk of bias.

### Evaluation of the outcomes

Modified USPHS criteria were used in 57.1% of the reviewed studies [8, 21–23], while Vanherle criteria [24] were used in 42.9% of the studies [9, 19, 20]. The criteria of evaluation of Peumans et al. [20] were not clear, so the author was contacted, and he clarified the usage of Vanherle criteria in these two studies [9, 20].

### Study designs

Studies were randomized clinical trials (RCTs), and among the reviewed trials, three (42.8%) of them used split-mouth design [8, 21, 23], and four (57.2%) [9, 19, 20, 22] used parallel design. Some reviewed clinical trials [19–23] (71.4%) mentioned the inclusion/exclusion criteria. However, Van Dijken [8] did not show the exclusion criteria. Van Landuyt et al. [9] did not clearly represent the eligibility criteria for subject selection. In addition, five (71.4%) of the included studies [8, 19, 21–23] indicated that ethical approval was obtained from an appropriate institute, while the remaining two studies [9, 20] (28.6%) did not clearly indicate the ethical-related issues. Isolation was done by cotton rolls and saliva ejectors in all studies; sometimes wooden wedge, retraction cord, and transparent cervical matrix system were used. Rubber dam was not used in any study.

### Interventions in included studies

HEMA-free adhesive systems in all studies were one-step self-etch adhesives except Van Dijken [8]; there were two HEMA-free adhesives: one was one-step self-etch and the other was three-step etch and rinse. On the other hand, HEMA-containing adhesive systems had different applications. Moreover, only one type of composite restoration was used with all adhesives in these studies [9, 19–22]. However, different types of composite restorations were used in Van Dijken [8, 23]. All the included studies evaluated the clinical performance and of HEMA-free adhesive systems. In these studies [8, 22], extra monomers (MDP and TEGDMA) respectively were evaluated, and in Van Dijken [8], HEMA/TEGDMA-free adhesive system was evaluated. Oliveira et al. [22] evaluated the clinical performance of self-etching adhesive system associating the functional monomer HEMA and 10-MDP.

### Effectiveness of individual studies

#### Functional properties

##### Retention

Retention rate was measured either by modified USPHS criteria [22] taking Alpha or Charlie scores, or by Vanherle criteria [24, 25] with 1 or 2 score. According to the reviewed studies, five of them (71.4%) [9, 19–22] showed no significant difference between HEMA-free and HEMA-containing adhesive systems, while Van Dijken [8] revealed significant differences between the two types of adhesive systems. However, retention was not used as an item for evaluation in Van Dijken [23].

In the study by Moretto et al. [19], the retention rate was 98.8% and 93.8% for HEMA-free and HEMA-containing adhesives, respectively (*p* = 0.14); no significant difference was found between the two types of adhesives. In the study by Van Landuyt et al. [9], both adhesives had high retention rates, and no statistically significant differences could be shown. Additionally, large and sclerotic lesions were a statistically significant risk factor according to statistical GEE analysis. In the study by Peumans et al. [20], the overall retention rate did not be affected by the degree of sclerosis. After 9 years, the retention rate was 89.7% for both adhesives (*p* > 0.05).

In the study by Tekce et al. [21], the statistical analysis reported no significant difference between the HEMA-free and HEMA-containing adhesive systems with or without the surface sealant with regard to retention. Besides, *P*-value was 1 for the two types of adhesives with or without surface sealant between baseline and 2-year follow-up. Therefore, no significant difference existed. In Oliveria et al. [22] after 1-year follow-up, the retention of the restorations showed no statistical difference between the groups (*p* = 1.0000). After a 2-year follow-up, there was no statistical difference between the groups (*p* > 0.05). In the study by Van Dijken [8] (*p* < 0.05), there was a significant difference between the two types of adhesive systems.

##### Marginal adaptation

Marginal adaptation parameter was assessed either by modified USPHS criteria [21, 22] taking (Alpha or Bravo or Charlie or Delta) scores, or by Vanherle criteria [24, 25] taking scores from 1 to 5. Marginal adaptation parameter was controversial; two studies [19, 21] (28.5%) showed no significant difference between HEMA-free and HEMA-containing adhesive systems, while three studies [9, 20, 22] (42.8%) revealed that HEMA-free adhesive systems had significantly more marginal defects than HEMA-containing ones. Moreover, two studies [8, 23] (28.5%) did not make a statistical analysis for this parameter.

In the study by Moretto et al. [19], only one HEMA-free restoration (1.2%) showed a significant clinically unacceptable marginal defect at dentin margin, and one (HEMA-containing) restoration showed caries recurrence. The overall clinical success rate for 3 years was 92.6% for HEMA-containing and 97.6% for HEMA-free. This difference was not statistically significant (*p* = 0.16). After 3 years of clinical evaluation, both adhesives showed an increase in the percentage of minor, yet clinically acceptable marginal defects at the enamel side more so than the dentin side. In the study by Van Dijken [8], the marginal defect rate was 7.9% for HEMA-free and 27.1% for HEMA-containing after 5-year follow-up, while another study by Van Dijken [23] showed marginal defect was 6.6% for HEMA-free, and 11.8% for HEMA-containing after 6 years clinical evaluation. In the study by Van Landuyt et al. [9], the restoration margins kept to disintegrate, and after 5 years, only a few numbers of the restorations had excellent margins. The number of marginal defects in restorations with HEMA-free adhesives was twice higher compared to those in restorations with HEMA-containing adhesives. Regarding dentin margins, there was no difference between the adhesives. Most marginal defects were regarded as clinically acceptable and did not need any kind of intervention.

In the study by Peumans et al. [20], 3 HEMA-free and 16 HEMA-containing restorations showed no marginal defects, causing a significant difference (*p* = 0.0031). There were significantly more marginal enamel defects in the HEMA-free group. The number of minor, yet clinically acceptable marginal defects on the enamel side showed the biggest variation. There was no statistically significant difference between the two groups for severe enamel defects (*p* > 0.9999). Overall, there was no significant difference (*p* > 0.05). In the study by Tekce et al. [21], statistical analysis revealed no significant difference between the HEMA-free and HEMA-containing adhesive systems with or without the surface sealant with regard to marginal adaptation. In Oliveira et al. [22], HEMA-free, MDP-containing adhesive showed more significant marginal deficiency when compared with HEMA-containing, MDP-containing adhesive (*p* = 0.0376) and HEMA-containing, MDP-free adhesive (*p* = 0.0433). Only 13 restorations (65%) in HEMA-free, MDP-containing adhesive retained the Alpha rating at the first year of follow-up. On the contrary, in the marginal adaptation analysis after a 2-year follow-up, all groups differed statistically from each other. Even though, HEMA-free, MDP-containing adhesive revealed the highest significant deficiency, as only eight restorations (40%) retained the Alpha rating [22].

### Aesthetic properties

#### Marginal discoloration

Marginal discoloration parameter was measured either by modified USPHS criteria [22] taking Alpha or Bravo or Charlie scores, or by Vanherle criteria [24, 25] taking scores from 1 to 3. Among the reviewed clinical trials, four studies [19, 20, 22, 23] (57.1%) showed significant differences between the two types of adhesive systems, two of them [19, 23] revealed that the HEMA-containing adhesive systems had more significant marginal discoloration, while the other two studies [20, 22] showed that the HEMA-free adhesive systems had more marginal staining. Oliveira et al. [22] showed HEMA-free, MDP-containing adhesive system had more marginal staining than HEMA-containing, MDP-containing adhesive system. On the other hand, Van Landuyt et al. [9] and Tekce [21] reported no significant difference between them. Besides, Oliveira et al. [22] showed no significant difference between HEMA-free, MDP-containing adhesive and HEMA-containing, MDP-free adhesive systems. One study [8] did not make a statistically analysis for this parameter.

In the study by Moretto et al. [19], small superficial marginal discoloration was noticed at 32.9% of the HEMA-containing restorations and 26.8% of the HEMA-free restorations. The marginal discolorations were associated with the existence of minute marginal defects. Additionally, smokers had a statistically significant higher prevalence of localized marginal discoloration (GEE, *p* = 0.0059). It is interesting that, when patients with a history of smoking were taken into account, a considerably higher prevalence of marginal discoloration was seen in the HEMA-containing group than in the HEMA-free group (*p* = 0.0229). In the study by van Dijken [23], both groups showed minimal marginal discoloration, which was significantly higher for HEMA-containing adhesive system. In the study by Van Dijken [8], after a 5-year follow-up, the marginal discoloration rate was 17.2% for the HEMA-free adhesive and 8.6% for the HEMA-containing adhesive.

In the study by Van Landuyt et al. [9], HEMA-free adhesive showed marginally higher discoloration, but not significantly more than HEMA-containing adhesive after Bonferroni correction. In the study by Peumans et al. [20], no marginal discoloration was noticed in 28.2% of the HEMA-free adhesive restorations and 43.6% of the HEMA-containing adhesive restorations, causing a significant difference in favour of HEMA-containing adhesive (*p* = 0.01). Superficial localized marginal discoloration was noted in 59.6% and 44.2% of the HEMA-free and HEMA-containing restorations, respectively (*p* > 0.05). Restorations that were HEMA-free (52.1%) had a significantly greater rate of superficial marginal discoloration on the enamel side than those that were HEMA-containing (30.8%) (*p* = 0.0082). On the cervical dentin side, the percentage of superficial marginal discoloration was lower and relatively similar in both groups: 36.8% for HEMA-free and 38.5% for HEMA-containing (*p* > 0.05). Nine HEMA-free (8.7%) and 7 restoration HEMA-containing (6.7%) failed as a result of severe generalized marginal discoloration.

In the study by Tekce et al. [21], the statistical analysis reported no significant difference between the HEMA-free and HEMA-containing adhesive systems with or without the surface sealant with regard to marginal discoloration. In the study by Oliveira et al. [22], the marginal staining rates were higher in HEMA-free, MDP-containing adhesive than in HEMA-containing, MDP-containing (*p* = 0.0301), as only 13 restorations (65%) in HEMA-free, MDP-containing adhesive retained the Alpha rating at the first year follow-up. However, there was no significant difference between HEMA-free, MDP-containing and HEMA-containing, MDP-free for marginal staining (*p* = 0.0532). Furthermore, after 2-year follow-up, HEMA-free, MDP-containing and HEMA-containing, MDP-free revealed the greater marginal discoloration rate among the three groups (*p* < 0.05), as only 12 restorations (65%) each in them retained the Alpha rating. However, there was no significant difference between HEMA-free, MDP-containing and HEMA-containing, MDP-free (*p* = 0.0231).

#### Colour match

Colour match was measured according to modified USPHS criteria [21] taking Alpha or Bravo or Charlie scores. Three (42.8%) of the seven included trials [8, 21, 23] used colour match as a parameter of evaluation. Moreover, one trial [23] showed there was a significant difference between the two types of adhesive systems HEMA-free and HEMA-containing. Another one [21] revealed there was no significant difference with regard to this parameter, and the last one [8] did not have statistical analysis for this parameter**.**

In the study by Van Dijken [23], for both resin composites, a substantial drop in colour match was seen between baseline and 6 years (*p* < 0.05). The observed colour changes fell within the accepted score range, and there was a significant difference between the materials. In the study by Van Dijken [8] after 5-year follow-up, colour match rate was 27.6% for HEMA-free adhesive and 37.1% for HEMA-containing adhesive. This was the score rate of very good colour match. In the study by Tekce et al. [21], with regard to colour matching, only the HEMA-free adhesive used in addition to surface sealant displayed statistically significant differences (*p* = 0.015) between baseline and 1-year rates, besides between baseline and 2-year rates.

### Biological properties

#### Hypersensitivity

Hypersensitivity parameter was assessed either by modified USPHS criteria [22] taking Alpha or Charlie scores, or by Vanherle criteria [24, 25] taking scores from 1 to 2. From the included studies, three studies(42.8%) [19, 20, 22] showed there was no significant difference between the two types of adhesive systems HEMA-free and HEMA-containing, while these three studies [8, 9, 23] did not show statistical analysis of this parameter. However, they revealed the sensitivity was diminished in the two adhesive systems. On the other hand, Tekce et al. [21] did not evaluate the restoration with regard to teeth sensitivity.

In the study by Moretto et al. [19], baseline data showed low rates of tooth sensitivity 4.9% for HEMA-containing adhesive and 16.7% for HEMA-free adhesive, and it stayed stable during the period of a 3-year study. There were no significant differences between them. In the study by Van Dijken [23], none of the two groups had post-operative sensitivity. In the study by Van Dijken[8], no post-operative sensitivity was recorded by the participants. In the study by Van Landuyt et al. [9], sensitivity was further diminished for both adhesives. In the study by Peumans et al. [20], when compared to the 5-year recall, both groups showed a small increase in tooth sensitivity: 7.7% for both HEMA-free and HEMA-containing adhesives (*p* > 0.05). In the study by Oliveira et al. [22], only HEMA-free, MDP-containing presented post-operative sensitivity (*n* = 2) at the first year follow-up, but no statistical difference existed between it and the other two groups that contain HEMA (*p* = 0.2436 for both). After a 2-year follow-up, only HEMA-free, MDP-containing and HEMA-containing, MDP-free reported post-operative sensitivity (*n* = 1 in each group), but did not differ statistically from HEMA-containing, MDP-containing (*p* > 0.05).

#### Caries and tooth vitality

Three [20–22] (42.8%) of the included studies showed no significant difference between the two adhesive systems according to the parameter of caries occurrence. The remaining studies reported absence or lower level of secondary caries but without statistical analysis. Besides, none of the reviewed studies made caries risk assessment except Van Dijken [23], in which carries were estimated by means of clinical and sociodemographic information routinely available at the annual clinical examinations, e.g. incipient caries lesions and former caries history. Moreover, bite-wing radiograph was also used.

In the study by Moretto et al. [19], the absence of caries recurrence and tooth vitality were nearly 100% for both adhesives. In the study by Van Dijken [8], no secondary caries was seen around the restorations during the follow-up. In the study by Van Dijken [23], there were only two secondary caries lesions found, one in each group and affecting both patients at high caries risk. It was challenging to claim that any adhesive systems had superior caries-inhibiting characteristics because of the low caries frequency seen in both groups.

In the study by Van Landuyt et al. [9], no teeth lost their vitality as a result of the restorative procedure. Marginal caries did not cause for any restorations to be replaced or repaired. Although one patient had localized demineralization (“white spot”) within one restoration, there was no extra treatment than plaque removal that was necessary. In the study by Peumans et al. [20], only two restorations in the same patient, one in each group, failed due to marginal caries after 9 years (*p* > 0.05). In the study by Tekce et al. [21], there was no significant difference between the two types of adhesives with or without the sealant in caries recurrence between the baseline and 2-year follow-up (*p* > 0.05). In the study by Oliveira et al. [22], no secondary caries was observed in any of the groups at the first year or the second year follow-up (*p* = 1.0000).

## Discussion

The most reliable way to evaluate treatments is through randomized clinical trials, which help healthcare professionals to make decisions [26, 27]. Systematic reviews summarize the results from these trials and provide the best clinical evidence. The clarity of reporting in systematic reviews helps readers assess the strengths and weaknesses of these reviews [28]. According to the findings of the current systematic review, HEMA-free adhesive restorations had high retention rates similar to the HEMA-containing ones. However, their clinical performance with regard to marginal adaptation and discoloration is still controversial.

Retention is a reliable way to evaluate the effectiveness of adhesive systems, and it is a direct measure of the ability of the adhesive to hold the restoration in place [29]. The majority of studies [9, 19–22] showed that there was no significant difference between HEMA-free and HEMA-containing adhesive systems, and this result agreed with the systematic review by Silva et al. [4]. The good adhesion of HEMA-free adhesives may be attributed to functional monomers that facilitate additional primary chemical bonding, which appeared to be more beneficial than the micromechanical retention [20]. On the other hand, Van Dijken [8] reported that there was a significant difference, and HEMA-free adhesive systems had better retention. However, Van Dijken’s study had some drawbacks, which made the increased retention rate could not be attributed exclusively to HEMA. For example, there was a difference in the application method between the two types of adhesive systems (self-etching HEMA-free adhesive and 3-step etch and rinse HEMA\TEGDMA free adhesive) used in comparison with 2-step etch and rinse HEMA-containing adhesive. Moreover, no patient was excluded because of caries activity, periodontal condition, or parafunctional habits.

Marginal defects and marginal discoloration can deteriorate the seal of restoration, and they are common reasons for replacing or repairing restorations [4]. Three studies [9, 20, 22] (42.8%) showed there was a significant difference, and HEMA-free adhesive systems had more enamel marginal defects. Two of these studies [9, 20] used mild self-etch HEMA-free adhesive systems in comparison with 3-step etch and rinse HEMA-containing adhesive systems. As we know, mild self-etch adhesives produce a very shallow enamel etching pattern with reducing micro retention for infiltrated resin, as compared to phosphoric acid etched enamel [30] leading to more enamel marginal defects and stains. [25, 31, 32] The other third study [22] showed the marginal defect was more prone to HEMA-free adhesive systems because of the phase separation, which led to nanoleakage and marginal defect. These outcomes disagreed with Moretto et al. [19] and Tekce et al. [21] studies, which showed no significant difference between the two adhesive systems. Overall, the majority of marginal defects were clinically acceptable on the enamel side.

According to marginal discoloration, the majority of studies [19, 21–23] showed there was significant difference between the two types of adhesive systems. Two of them [19, 23] revealed HEMA-containing adhesive systems had more significant marginal discoloration, and there was some concern in the methodology of these studies; in Van Dijken [23], no patient was excluded because of oral hygiene, caries activity, periodontal condition, or parafunctional habits. Van Dijken study used (class II) cavity design without the application of rubber-dam. Consequently, that can affect the results, since a meta-analysis by Mahn et al. [33] showed rubber dam application decrease the possibility of marginal discoloration in cervical restorations. In Moretto et al. [19], the study included smoker patients that can influence the outcome of marginal discolorations. In Oliveira et al. [22], there was confliction in results with respect to this parameter because it was a three-arm comparative study; marginal staining was significantly higher in HEMA-free, MDP-containing adhesive system than in HEMA-containing, MDP containing adhesive system. On the other hand, there was no significant difference between HEMA-free, MDP-containing adhesive system and HEMA-containing, MDP-free adhesive system. That means the functional monomer MDP had an effect on marginal discoloration.

The incidence of recurrent caries was dropped in all studies; the possible explanation was that NCCLs, which were used in most studies, have limited variations in shape and are easy to clean, because they were self-cleansing regions with visual accessibility. This helps to keep the surface free of biofilm, thus decreasing the risk of caries [4, 34]. In the majority of studies, there was no post-operative sensitivity from the two types of adhesive systems especially in NCCLs, because the NCCLs have a good C-factor and do not cause a lot of stress at the interface [4].

This current systematic review aimed to evaluate the clinical performance of HEMA-free adhesive systems in any cavity design, and it included previous randomized clinical trials with at least 2-year clinical follow-up. The aim with sufficient follow-up period was to evaluate the durability of interfacial bond over time and to evaluate the effect of exposure to oral fluids, chemical and physical stress factors like chewing loads, pH, and temperature changes [35]. Studies done between 2013 and 2023 were also included for the reason of the development and availability of materials. There were many excluded studies such as studies done on endodontically treated teeth, which had higher failure rates than vital ones [36], and on primary teeth as they had lower bond strength and worse adhesion than permanent teeth [37]. Trials with one-arm comparative study were excluded too such as Van Dijken et al. [38].

In this current systematic review, most of studies [8, 9, 19, 20, 22] used non-carious cervical lesions (NCCLs) to evaluate the effectiveness of adhesives, because NCCLs do not have a lot of natural retention. Hence, the adhesive is the only thing holding the restoration in place [20]. On the other hand, the study by Van Dijken [23] used class II cavities, because the dentin of NCCLs does not reflect the substrate of other cavity designs especially class II cavities. Moreover, adhesive systems need to be tested in load-bearing posterior cavities [39]. One point to be mentioned is the method of isolation, all trials did not use rubber dams in isolation just cotton rolls, an aspirator, and wooden wedges. A transparent matrix and retraction cord may be used. A meta-analysis by Mahn et al. [33] revealed that rubber-dam isolation had a positive influence on the retention rate of the restorations. However, other studies showed that the use of cotton rolls, retraction cord, or rubber dam resulted in similar clinical data in terms of retention rate in non-carious cervical lesions [40]. For the quality of evidence, the assessment of certainty of evidence depends on many principle factors.

- First: The risk of bias. Six included studies [8, 9, 19–21, 23] showed unclear risk of bias, while Oliveria et al. [22] showed high risk of bias.
- Second: The precision of the effect estimates and the consistency of the individual study results, which were not applicable to determine for the included trials in this systematic review owing to the heterogeneity in their study design, follow-up periods, and assessment criteria. Thus it was not possible to perform a quantitative analysis (meta-analysis).
- Third: How directly the evidence answers the question of interest. Unfortunately, the evidence did not completely answer the research question and was not enough to support the performance of HEMA-free adhesives on marginal adaptation and marginal discoloration. Thus, the results need to be confirmed with long-term evaluations.
- Fourth: The risk of publication or reporting biases. Two included studies [21, 22] registered at clinical trials, and their protocol coincides with their final outcomes. Thus, their reporting bias was low.

The remaining included studies [8, 9, 19, 20, 23] did not mention their registration at clinical trials, and it was difficult to assess their reporting bias. The limitations of this review were different adhesive approaches and methodologies, wide patient age range, and short follow-up period in some studies that led to little evidence. Furthermore, heterogeneity in study design and ignoring rubber dam isolation must be taken into consideration.

## Conclusion

HEMA-free adhesive systems exhibited good clinical performance with regard to retention. The current level of evidence was not enough to support their performance on marginal adaptation and marginal discoloration, as a result of conflicted results of the included trials. Thus, the results need to be confirmed with long-term evaluations.

## Data Availability

The datasets generated during and/or analyzed during the current study are available from the corresponding author on reasonable request.
